# Effects of Pneumococcal Conjugate Vaccine 2 Years after Its Introduction, the Netherlands

**DOI:** 10.3201/eid1605.091223

**Published:** 2010-05

**Authors:** Gerwin D. Rodenburg, Sabine C. de Greeff, Angelique G.C.S. Jansen, Hester E. de Melker, Leo M. Schouls, Eelko Hak, Lodewijk Spanjaard, Elisabeth A.M. Sanders, Arie van der Ende

**Affiliations:** Wilhelmina Children’s Hospital/University Medical Center, Utrecht, the Netherlands (G.D. Rodenburg, A.G.C.S Jansen, E. Hak, E.A.M. Sanders); National Institute for Public Health and the Environment, Bilthoven, the Netherlands (S.C. de Greeff, H.E. de Melker, L.M. Schouls); University Medical Center, Groningen, the Netherlands (E. Hak); Netherlands Reference Laboratory for Bacterial Meningitis, Amsterdam, the Netherlands (L. Spanjaard, A. van der Ende); ^1, 2^These pairs of authors contributed equally to this study.

**Keywords:** Heptavalent pneumococcal conjugate vaccine, pneumococcal vaccines, pneumococcal infections, surveillance, bacteria, research

## Abstract

In the Netherlands, the 7-valent pneumococcal conjugate vaccine (PCV-7) was implemented in a 3+1-dose schedule in the national immunization program for infants born after April 1, 2006. To assess the vaccine’s effectiveness, we compared disease incidence before and after vaccine implementation (June 2004–June 2006 and June 2006–June 2008, respectively). We serotyped 2,552 invasive pneumococcal isolates from throughout the Netherlands, covering 25% of the country’s population. Clinical characteristics were extracted from hospital records. After June 2006, vaccine-serotype invasive pneumococcal disease (IPD) decreased 90% (95% confidence interval [CI] 68%–97%) in children age eligible for PCV-7; simultaneously, however, non–vaccine-serotype IPD increased by 71% (not significant), resulting in a 44% total net IPD reduction (95% CI 7%–66%). IPD rates did not change for other age groups. In the Netherlands, PCV-7 offered high protection against vaccine-serotype IPD in vaccinated children, but increases of non–vaccine-serotype IPD reduced net vaccine benefits.

## Abstract 2

Vaccine-serotype disease decreased, but non–vaccine-serotype disease increased.

*Streptococcus pneumoniae* is a leading cause of invasive infections, such as meningitis, septicemia, and bacteremia, and of more common respiratory tract infections, such as pneumonia and otitis media. Young children and elderly persons are at particularly high risk for pneumococcal infection (*1*). In the United States, the introduction in 2000 of the CRM197-conjugated 7-valent pneumococcal vaccine (PCV-7) resulted in a 77% reduction in 2005 of invasive pneumococcal disease (IPD) in children <5 years of age from IPD rates reported in 1998–1999 (*2*). IPD rates in children decreased mostly within the first 2 years after introduction of PCV-7; leveled off in 2002; and then stabilized, despite an ongoing decrease of vaccine-serotype IPD, due to a gradual increase of non–vaccine-serotype IPD, particularly serotype 19A (*2**,**3*). In addition, use of the vaccine in children was associated with reduced IPD rates for unvaccinated age groups, which resulted from reduced nasopharyngeal colonization of vaccine-serotype *S. pneumoniae* in vaccinated children and concomitant reduced transmission (*4**,**5*). The cost effectiveness of herd immunity conferred by the conjugate vaccine in the United States prompted implementation of the vaccine in the Netherlands (*6*).

Data from the United States concerning both direct and indirect vaccine benefit, however, cannot be translated indiscriminately to European countries because of several major differences. Vaccine-serotype coverage by PCV-7 was lower in European countries (60%–70%) than in the United States (>80%) (*7*), which may leave more room for non–vaccine-serotype replacement in European countries. Second, in the Netherlands (as in most European countries), baseline IPD incidence rates are based mainly on culture-confirmed cases in hospitalized children, resulting in markedly lower IPD incidence rates for young children in the Netherlands than for those in the United States, where blood samples are cultured for more patients. Before introduction of PCV-7 in the Netherlands, overall IPD rates were 35 cases/100,000 children <2 years of age, of which 15 cases/100,000 children were meningitis (*1*). In contrast, in the United States, IPD incidence before introduction of PCV-7 peaked at 188 cases/100,000 children <2 years of age in 1998–1999 (*5*), and 10 cases/100,000 children in that age group were meningitis (*8*).

Consequently, introduction of PCV-7 may have affected IPD incidence in European countries differently than in the United States. (*9*). To assess the effectiveness of PCV-7 on IPD in the Netherlands, we evaluated the incidence and clinical syndromes of IPD in PCV-7–vaccinated and –unvaccinated children and in other age groups during the first 2 years after implementation of PCV-7.

## Materials and Methods

### Surveillance and Data Collection

PCV-7 was introduced into the Netherlands’ national immunization program (NIP) in June 2006 and was recommended for all infants born after April 1, 2006, at 2, 3, 4, and 11 months of age (*10*). Our study comprised all patients with culture-confirmed IPD during June 1, 2004–June 1, 2006 (preimplementation period) and June 1, 2006–June 1, 2008 (postimplementation period). Isolates were serotyped by the Netherlands Reference Laboratory for Bacterial Meningitis (NRLBM), which collects nationwide bacterial isolates from blood, cerebrospinal fluid (CSF), and/or other normally sterile bodily fluids for laboratory-based surveillance. Isolates from all patients with IPD were submitted by 9 sentinel laboratories throughout the country. These laboratories covered ≈4.074.412 and ≈4.090.233 residents in the preimplementation period and postimplementation period, respectively, representing ≈25% of the population of the Netherlands. Laboratories were selected on the basis of their reliability for submitting pneumococcal isolates; they submitted ≈90%–95% of the pneumococcal isolates from CSF and ≈83% of pneumococcal isolates from blood (*1*).

Isolates were serotyped as previously described, by using antiserum from the Statens Serum Institute (Copenhagen, Denmark) (*1*). Isolates with serotypes 4, 6B, 9V, 14, 18C, 19F, and 23F, the serotypes contained in PCV-7, were considered vaccine serotypes. All other serotypes were considered non–vaccine-serotypes.

### Clinical Characteristics

Nearly all (97%–98%) IPD cases were in hospitalized patients. We retrospectively abstracted information about their clinical syndromes and underlying conditions from hospital records. Clinical syndromes were categorized as meningitis or nonmeningitis IPD (invasive pneumonia, IPD with other focus, and bacteremia without focus). Meningitis was defined as CSF culture positive for *S. pneumoniae* (or positive CSF by PCR) and/or clinical diagnosis of meningitis in combination with a blood culture positive for *S. pneumoniae*. Invasive pneumonia was physician-diagnosed pneumonia and a blood culture positive for *S. pneumoniae*. IPD with other focus was an *S. pneumoniae*–positive culture of blood or other normally sterile body fluid in combination with a clinical focus other than meningitis or pneumonia. For bacteremia without focus, no clinical focus was identified. Underlying conditions were classified as immunocompromised conditions or other comorbidities, as described previously (*1*). Case fatality was defined as in-hospital death and/or death within 30 days after the first reported blood/CSF culture positive for *S. pneumoniae*.

### Statistical Analyses

To study the effectiveness of the vaccination program, we compared age-specific incidences during the preimplementation and postimplementation periods. Incidence rates of IPD were calculated as number of cases per 100,000 persons per year by using 25% of the Dutch population on January 1 for each considered year, accounting for the 25% coverage of surveillance data. Changes in incidence rates from the preimplementation to the postimplementation period were presented as incidence rate ratio with 95% confidence intervals (CI) and as percent changes. We compared the preimplementation and postimplementation periods with regard to distribution of clinical syndromes, comorbidities, and outcomes. Theoretical coverage of IPD was based on data from the preimplementation and postimplementation periods for future 10-valent PCV (PCV-10, covering PCV-7 serotypes plus serotypes 1, 5, and 7F) and 13-valent PCV (PCV-13, covering PCV-10 serotypes plus serotypes 3, 6A, and 19A). Proportions were tested with χ^2^ or Fisher exact tests, as appropriate. We considered p <0.05 to be significant. Statistical analyses were performed with SAS version 9.1.3 (SAS Institute, Cary, NC, USA), Excel 2007 (Microsoft, Redmond, WA, USA), and Episheet (*11*).

## Results

During the study period, the NRLBM received 2,649 *S. pneumoniae* isolates: 1,297 during the preimplementation period and 1,352 during the postimplementation period. Medical records were assessed for 1,235 (95%) cases during the preimplementation period and for 1,317 (97%) cases during the postimplementation period. Pneumococcal serotype was available for 1,225 and 1,304 cases (both 99%), respectively.

### IPD Incidence

Overall incidence of IPD remained stable; 15.9 vs. 15.0 cases/100,000 persons during the postimplementation and preimplementation periods, respectively. Incidence of vaccine-serotype IPD did not change significantly. For non–vaccine-serotype IPD, incidence increased 13% (95% CI 2%–26%; p = 0.02) (Table). In children <2 years of age, including those not vaccinated or incompletely vaccinated, the incidence of IPD decreased 35% (95% CI 4%–56%; p = 0.006), from 34.5 cases/100,000 persons in the preimplementation period to 22.5 cases/100,000 persons in the postimplementation period (Table). Incidence of vaccine-serotype IPD declined by 67% (95% CI 41%–81%; p<0.0001), from 24.3 to 8.0 cases/100,000 persons. In contrast, non–vaccine-serotype IPD incidence increased, but not significantly, from 10.1 to 14.5 cases/100,000 persons (p = 0.40).

**Table:** Incidence rates of invasive pneumococcal diseases before and after implementation of 7-valent pneumococcal conjugate vaccine, the Netherlands*

Serotypes by patient age group, y	Preimplementation period (June 2004–June 2006)			Postimplementation period (June 2006–June 2008)			Preimplementation vs. postimplementation	
	No. cases	Rate		No. cases	Rate		IRR (95% CI)	p value†
Total								
All ages	1,225	15.0		1,304	15.9		1.06 (0.98–1.15)	0.14
<2	68	34.5		42	22.5		**0.65 (0.44–0.96)**	**0.006**
2–4	25	8.1		26	8.7		1.07 (0.62–1.86)	
5–49	206	4.1		231	4.7		1.13 (0.94–1.37)	
50–64	254	16.7		292	18.5		1.11 (0.94–1.31)	
>65	672	58.8		713	60.2		1.02 (0.92–1.14)	
Vaccine serotypes‡								
All ages	570	7.0		561	6.9		0.98 (0.87–1.10)	
<2	48	24.3		15	8.0		**0.33 (0.19–0.59)**	**<0.0001**
2–4	17	5.5		17	5.7		1.03 (0.53–2.02)	
5–49	69	1.4		70	1.4		1.02 (0.73–1.43)	
50–64	114	7.5		129	8.24		1.09 (0.85–1.40)	
>65	322	28.2		330	27.9		0.99 (0.85–1.15)	
Nonvaccine serotypes§								
All ages	656	8.0		743	9.1		**1.13 (1.02–1.26)**	**0.02**
<2	20	10.1		27	14.5		1.43 (0.80–2.55)	
2–4	8	2.6		9	3.0		1.16 (0.45–3.01)	
5–49	137	2.8		161	3.3		1.19 (0.94–1.49)	
50–64	140	9.2		163	10.3		1.12 (0.89–1.41)	
>65	350	30.6		383	32.4		1.06 (0.91–1.22)	

*Rate is cases/100,000 persons. IRR, incidence rate ratio; CI, confidence interval. **Boldface** indicates significant differences (p<0.05). †p values shown <0.15; incidence rates pre vs. postimplementation period. Calculated by using Fisher exact test; all p values are 2 sided. ‡*Streptococcus pneumoniae* serotypes 4, 6B, 9V, 14, 18C, 19F, and 23F. §All other *S. pneumoniae* serotypes.

**Figure 1:**
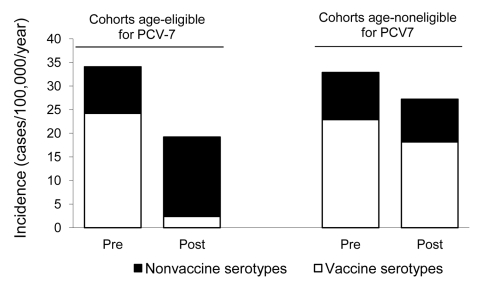
Incidence of invasive pneumococcal disease in children <2 years of age in the birth group born after April 1, 2006 (age eligible for 7-valent pneumococcal conjugate vaccine [PCV-7]) and children born before April 1, 2006 (age noneligible for PCV-7), in the postimplementation period compared with age-matched children in the preimplementation period, the Netherlands. Incidence per 100,000 children <2 years of age per year; Pre, preimplementation period (June 2004–June 2006); post, postimplementation period (June 2006–June 2008).

Among children born after April 1 2006 (i.e., age-eligible for vaccination according to the NIP), the incidence rate of vaccine-serotype IPD in the postvaccination period (2.4 cases/100,000 persons) decreased 90% (p<0.0001) compared with that for an age-matched group in the preimplementation period (24.2 cases/100,000) (Figure 1). Although not significant because of low numbers, the incidence of non–vaccine-serotype IPD had risen by 71%, from 9.8 to 16.8 cases/100,000 persons (p = 0.12), leading to a total net reduction of 44% (95% CI 7%–66%; p = 0.02) in the birth group age-eligible for vaccination.

Three vaccine-serotype IPD cases occurred among children born after April 1, 2006; 2 cases after 1 vaccine dose (serotypes 9V and 23F) and 1 case within 1 week after the second dose (serotype 9V, isolated from CSF). In infants <2 years of age born before April 1, 2006 (i.e., age-ineligible for PCV-7), no changes occurred in vaccine- or non–vaccine-serotype IPD rates in the postimplementation period compared with those for age-matched children in the preimplementation period.

### Serotype Distribution

**Figure 2:**
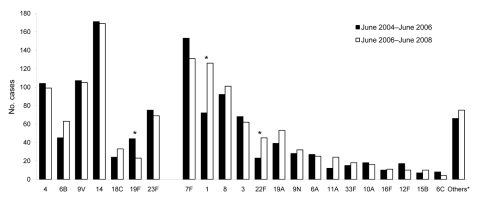
Serotype distribution of invasive pneumococcal disease with regard to preimplementation and postimplementation of 7-valent pneumococcal conjugate vaccine (PCV-7); among persons of all ages, the Netherlands. Preimplementation period June 2004–June 2006; postimplementation period June 2006–June 2008; *p<0.05; proportion of serotypes preimplementation vs. postimplementation period. Calculated using Fisher exact test; all p values are 2 sided.

**Figure 3:**
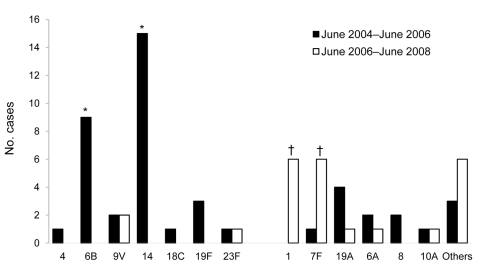
Serotype distribution of invasive pneumococcal disease cases among children born after April 1, 2006 (age eligible for 7-valent pneumococcal conjugate vaccine [PCV-7]) in the postimplementation period compared with age-matched children in the preimplementation period, the Netherlands. Preimplementation period, June 2004–June 2006; postimplementation period, June 2006–June 2008; other serotypes are 15A, 16F, 22F, 3, 33F, 5, and 9N. *p<0.05; preimplementation vs. postimplementation periods. Proportions calculated using Fisher exact test; all p values are 2 sided.

After introduction of PCV-7, for all vaccine serotypes, the number of IPD cases among the total population remained stable, except for serotype 19F (44 vs. 23 cases; p = 0.004). Also, proportions of non–vaccine-serotype 1 and 22F significantly increased (Figure 2). Among children born after April 1, 2006, serotypes 1 and 7F increased in comparison with those for age-matched infants in the preimplementation period (Figure 3). For serotype 6A, 6C and 19A, no significant changes occurred in any age group.

### Clinical Characteristics

**Figure 4:**
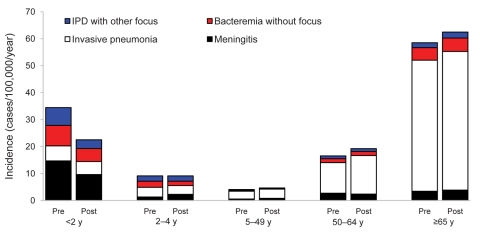
Age group–specific distribution of clinical invasive pneumococcal disease (IPD) syndromes in the preimplementation and postimplementation periods of 7-valent pneumococcal conjugate vaccine (PCV-7), the Netherlands. Incidence is IPD cases per 100,000 persons per year. Pre, preimplementation period (June 2004–June 2006); post, postimplementation period (June 2006–June 2008).

Among children <2 years of age, incidence rates decreased for all clinical syndromes to approximately the same extent (Figure 4). Rates of meningitis declined 34% from 14.7 to 9.6 cases/100,000 children (29 vs. 18 cases) and of nonmeningitis IPD 35% from 19.8 to 12.9 cases/100,000 children in this age group (39 vs. 24 cases). Of these children, 30% (20/66) had comorbidities in the preimplementation period, compared with 9% (4/44) in the postimplementation period (p<0.001). In all other age groups, clinical syndromes did not change from the preimplementation to the postimplementation period, except for a 124% rise in rates of non–vaccine-serotype meningitis for persons 5–49 years of age (95% CI 19%–320%; p = 0.01). The proportions of adult patients with comorbidities and immunocompromising conditions were similar in the preimplementation and postimplementation periods: 71% vs. 74% and 19 vs. 22%, respectively. For the vaccinated group of children, the case-fatality rate remained stable (9.3% vs. 8.3%) in the preimplementation and postimplementation periods, respectively. In other age groups, case-fatality rates did not differ (data not shown).

### Estimated Coverage by Future Vaccines

**Figure 5:**
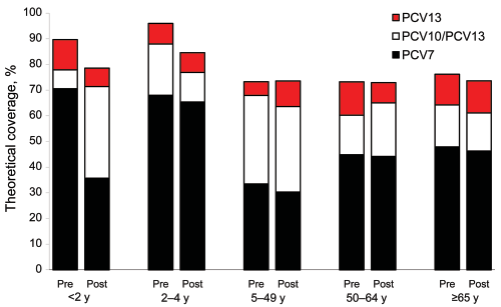
Age group–specific theoretical coverage of pneumococcal conjugate vaccines during the preimplementation and postimplementation periods of 7-valent pneumococcal conjugate vaccine (PCV-7), the Netherlands. IPD, invasive pneumococcal disease; PCV-10/PCV13, additional coverage by PCV-10 and PCV-13; PCV-13, additional coverage by PCV-13 alone; pre, preimplementation period (June 2004–June 2006); post, postimplementation period (June 2006–June 2008).

Among vaccination-eligible children, the additional coverage rates in the preimplementation and postimplementation periods were 2.2% (1/45 cases) and 54.2% (13/ 24 cases) for PCV-10 (p<0.0001). For PCV-13, they were 19.6% (8/45 cases) and 66.7% (16/24 cases) (p<0.001) (Figure 5).

## Discussion

The total net IPD reduction of 35% among children <2 years of age observed in the Netherlands differs from the favorable results reported in the United States, where a 69% decrease in IPD within the first 2 years after PCV-7 introduction was reported despite lower vaccine uptake (i.e., estimates of national immunization coverage) rates in the United States than in the Netherlands (*2**,**12*). US estimates for PCV-7 uptake among children born in 2001 who received >1 and >3 doses were 89% and 68%, respectively (*2*). In contrast, in the Netherlands, 94.4% of all infants born in 2006 were fully vaccinated at 2 years of age (*12*). Also, the decrease in meningitis incidence in the Netherlands was lower than that for US infants <2 years of age (34% vs. 59%), whereas during the preimplementation period, meningitis incidence was comparable in the 2 countries. In the first 2 years after implementation in the United States, IPD requiring hospitalization decreased 63% in children <2 years (*5*). In contrast, all IPD in this age group decreased 35%, and almost all reported IPD cases occurred in hospitalized patients.

The difference between the impact of PCV-7 in the Netherlands and the United States may be attributable to the lower proportion of vaccine-serotype cases covered by PCV-7 before implementation in the Netherlands and in Europe. Reports about PCV-7 effectiveness by other European countries support this observation. In Germany and Norway, PCV-7 effectively prevented disease in the youngest age groups during the first years after implementation, without major increase of non–vaccine-serotype IPD (*13**,**14*). In Spain, vaccine-serotype IPD decreased after PCV-7 implementation; however, non–vaccine-serotype IPD increased (*15**,**16*). In France, 3 years after PCV-7 introduction, overall IPD cases decreased 21% among children of <2 years of age, when 44%–56% of children were vaccinated (*17*). In addition, similar to findings in the Netherlands, a simultaneous increase in IPD from non–vaccine-serotype pneumococci reduced the net benefit of vaccination. Differences in surveillance systems, temporal fluctuations of circulating serotypes, antimicrobial drug resistance and penicillin susceptibility of circulating pneumococcal strains, vaccination schedules, vaccine uptake, and blood sampling practices also may play a role in the differences between countries (*9**,**18*). Like Norway, a reduced-dose PCV-7 schedule has been introduced in the United Kingdom. In the first years after implementation, surveillance data from the United Kingdom have tended to show a major decline in vaccine-serotype IPD in infants <2 years of age concomitant with a substantial rise in non–vaccine-serotype IPD, reducing net vaccine benefits (*19**,**20*). Unlike in the United States, where IPD in children <2 years of age stabilized within 2 years after introduction of the vaccine (*2*), in Europe and in our study, incidence has not yet stabilized. Our results emphasize the need for continued surveillance to monitor the long-term public health benefits of the vaccination program.

Despite the high vaccination uptake in the Netherlands, we observed no indication of herd immunity in other age groups during the first 2 years after implementation, except for a decrease in serotype 19F. This observation may be explained by the relatively short evaluation period of 2 years, a relatively small vaccinated group (2.25%) of the total population, and lack of a catch-up program for older children. In Australia and the United Kingdom, which have catch-up programs for children <2 years of age, decreases in vaccine-serotype IPD in unvaccinated children within 3 years after PCV-7 implementation have been reported (*21**,**22*).

The small increase we found in non–vaccine-serotypes 1 and 7F among vaccinated children could be attributed to temporal fluctuations (*18**,**23*). The numbers in our study were too small and the period we studied too short to enable us to draw firm conclusions about changes in serotype-specific incidence. Serotype 1 also has increased in other age groups and may cause local outbreaks, as observed in other countries before the implementation of PCV-7 (*24**,**25*). In our study, we could not find evidence of outbreaks associated with serotype 1.

The increase in non–vaccine-serotype IPD in the vaccinated age group was not explained by more children with comorbidities or immunocompromised conditions in the years after introduction and not associated with a change in the case-fatality rate. Longer follow-up is needed to assess whether this increase and that in non–vaccine-serotype IPD in the overall population are temporary or are vaccine related. Several countries have suggested that use of PCV-7 might enhance the emergence of serotype 19A pneumococcal clones, often associated with penicillin resistance (*26*). In the Netherlands, where use of antimicrobial drugs is restricted, few penicillin-resistant pneumococcal isolates were received during the study period; 98.8% were susceptible to penicillin (MIC <0.06 mg/L), 0.8% were intermediately susceptible (0.06–1.0 mg/L), and 0.4% were resistant (>1.0 mg/L). We did not see a prominent increase in serotype 19A among patients; only 1 isolate was penicillin resistant (>1.0 mg/L) in the first 2 years after PCV-7 implementation. However, an increase in serotype 19A pneumococci was found in a randomized controlled study of nasopharyngeal carriage among vaccinated children compared with unvaccinated controls before national implementation of PVC-7 (*27*). Also, no changes in distribution of serotype 6A or 6C were reported. Theoretical coverage of the future conjugate vaccines PCV-10 and PCV-13 increased in the postimplementation period in vaccination-eligible children. In all other age groups, no changes were observed. Future vaccines need to be considered to improve the net benefit of immunization against pneumococcal diseases.

Some limitations should be acknowledged. Although our study covered ≈25% of the population of the Netherlands, numbers of IPD cases and serotype distribution are small and need cautious interpretation. After 2 years, final vaccine benefits cannot be established. Furthermore, distribution of serotypes among invasive pneumococci may fluctuate over time, and temporal trends in serotype may vary across geographic regions and independent of PCV-7 implementation (*18**,**23*). Although the 9 sentinel laboratories submitted 25% of all pneumococcal isolates received by the NRLBM (nationwide coverage 95%), the population under surveillance might be overestimated because the laboratories were selected on reliability of stable pneumococcal isolates submission over the years. Second, our surveillance system depended on how well the 9 sentinel laboratories were submitting their isolates, and small shifts in the proportion of submission to the NRLBM cannot be excluded (*14*). Blood culture rates may have influenced IPD incidence reported in this study (*28*). However, these changes are not likely to be substantial. National IPD incidence rates estimated from the number of isolates submitted by the 9 sentinel laboratories are similar to those of neighboring countries, e.g., Denmark and the United Kingdom, that have comparable health system practices (*29*). The rates of submission of blood and CSF isolates for children <5 years of age have been stable in the Netherlands for the past 10 years at ≈90%. Enhanced surveillance with such high submission rates and stable overall IPD rates cannot explain the 71% increase of nonvaccine serotypes. Also, and most important, the changes in IPD serotype distribution occurred only in vaccination-eligible infants. No changes in serotype distribution or signs of herd immunity were observed in unvaccinated infants. A potential bias by enhanced awareness would cause differences also in this group. Long-term surveillance data will elucidate whether the changes in serotype distribution in vaccinated and unvaccinated persons remain. Lastly, in the Netherlands, no changes in diagnostic methods or blood culture practices have been implemented recently.

Strengths of our study include the detailed information about the IPD cases and the established high degree of vaccine uptake for the NIP in the Netherlands (≈95% of infants of <1 year of age are fully vaccinated) (*12*). Vaccination with the 23-valent pneumococcal polysaccharide vaccine has not been routinely recommended for elderly persons, and uptake has been negligible in the Netherlands; thus, any influence of this vaccine can be excluded (*30*). The low proportion of penicillin-resistant pneumococcal isolates and the densely living but relatively homogeneous population make the Netherlands particularly suitable for describing vaccine effects. Our study provides accurate data from a representative group of the Dutch population with fairly detailed information about the distribution of clinical syndromes and presence of comorbidities.

Shortly after introduction of PCV-7 vaccination for infants, the direct vaccine effectiveness on IPD caused by vaccine-serotype pneumococci appeared high in the Netherlands. However, the net benefit is partly offset by the increased incidence of nonvaccine serotypes. For this reason, future conjugate vaccines may be valuable in further reducing IPD incidence. These results further emphasize the need for ongoing surveillance.
